# What are the main motivating factors for young general practitioner trainees to work in rural areas in the Czech Republic?

**DOI:** 10.1080/13814788.2022.2094913

**Published:** 2022-07-07

**Authors:** Kateřina Javorská, David Halata, Josef Štolfa, Markéta Pfeiferová

**Affiliations:** a Working Group on Rural Practice of the Czech GP Society; b Department of Preventive Medicine, Charles University, Faculty of Medicine in Hradec Králové, Czech Republic; c Praktický lékař Javorský s.r.o., Nové Město nad Metují, Czech Republic; d VicusMedicus s.r.o, Hošt´álková, Czech Republic; e Institute for Postgraduate Health Education, Prague, Czech Republic; f Czech Young GPs, Czech Republic

**Keywords:** Motivating factors, young GPs, rural general practice

## Abstract

**Purpose:**

The global health workforce suffers long-term understaffing in remote and underserved areas. To attract young doctors for rural work, it is necessary to identify the main motivating factors.

**Materials and methods:**

The pilot survey with 201 general practitioner trainees in the Czech Republic was conducted using a structured questionnaire. The response rate was 67%.

**Results:**

Not only financial support motivates general practitioner trainees for rural work. A combination of incentives from sources other than medical would greatly increase the chance for general practitioner trainees to work in rural regions.

**Conclusions:**

To what extent can the survey outcomes relate with other European regions needs to be investigated further.

KEY MESSAGES

- The main motivating factors leading general practitioner trainees to work in rural areas in the Czech Republic are finance, securing a suitable job for a partner and securing schooling for children, ideally all three fulfilled.
- Contrary to common expectations, incentives shall be complex and focus on rural life in general.

One of the most difficult challenges for health systems in Europe is understaffing of rural surgeries [1]. The European Rural and Isolated Practitioners’ Association (EURIPA) represents a growing network of rural practitioners and national organisations across Europe working together to disseminate good practice, initiate research and influence policies [2]. This group is preparing a Europe-wide qualitative study of trainees in general practice (GP trainees) to find motivators and barriers to choosing careers in rural regions.

To assess the feasibility of this study, we recently performed a pilot survey among GP trainees in the Czech Republic. We approached 300 GP trainees (out of 645 in training that year) during a course conducted by the Institute for Postgraduate Health Education in Prague. A questionnaire was used to identify the main motivating factors (see Appendix). Out of 201 trainees that responded (response rate 67%), 145 were female and 60 did not have General Practice as their first career choice. Most trainees (48%) were considering a career as a rural GP, a small group of trainees (15%) had not decided yet, and more than one-third had decided against it (37%).

The main motivators identified are summarised in Table 1; number of respondents and motivating factors stated.

**Table 1.** Number of respondents and motivating factors.

Motivators	First choice	Requalifying	Men	Women
Finance	104	45	46	97
Job for a partner	90	34	38	95
Schooling for children	112	47	45	110

Most importantly, 62% of those contemplating a career in rural general practice stated that they would have definitely decided for it if all top three factors were fulfilled. The main concern about a rural practice was the accessibility to subsequent secondary care facilities. In addition, we found that 86% of respondents felt they did not have access to relevant information on rural practice and 70% of respondents would appreciate a short-term training period in a rural practice as a part of the general practice training programme.

Our pilot study surveying GP trainees provided excellent response rates. Obviously, our findings in about one-third of all GP trainees in a single country, lack generalisability and should be considered as preliminary. Nonetheless, it is evident that contrary to common expectations, financial incentives are not the single most important motivation for a career in rural general practice. A Europe-wide survey is currently underway and its results will help policy makers decide which recruitment and retention strategies as well as supporting measures are most efficient [3–5]. We are grateful and positive that attention is finally given to a field neglected for many years.

## Appendix

### What are the main motivating factors for young general practitioners to work in rural areas?

#### Czech Young GPs, Working Group on Rural Practice of the Czech GP Society, Institute for Postgraduate Health Education, Czech Republic

For those living in the country, a rural general practitioner is the primary (and often exclusive) link to healthcare. However, young general practitioners (GPs) in Europe are mostly associated with practices in large cities and a lack of rural GPs is emerging. Their absence in rural areas can consequently lead to inaccessibility of healthcare and the population’s suffering. Foreign experience points out this trend in many European Countries. What is the actual situation in the Czech Republic?

According to available data, rural healthcare is provided by 50% of all GPs in the Czech Republic. For this questionnaire, a rural area is defined as any district or settlement without a local hospital or a hospital accessible by a city public transportation system.

**Questionnaire**

**Sex – M – F**

**Age:**

**Year of graduation:**

**Requalifying from other specialities**

Yes – No; state the speciality if requalifying……………………………………….

**Medical faculty:**

**Region of origin (birth):**

1. **Do you live in a rural area?** Yes – No
2. **Do you work in a rural area at the moment?** Yes – No
3. **If you do not work in a rural area, would you consider working there?** Yes – No
4. **When considering the location of your future practice, which factors do you find most important?** (Select the five most important factors)
  - Income, profitability of the practice
  - Environmental issues
  - City life
  - Country life
  - Accessibility of schooling for your children
  - Accessibility of qualified jobs for your partner
  - Accessibility of culture (theatres, cinemas or others)
  - Accessibility of sport facilities
  - Desire to work where you grew up
  - Others – specify:
5. **Is one-time financial support from the Ministry of Health for a GP practice around 300,000 – 500,000 Czech Crowns motivational for you to decide to work in rural areas?** Yes – No
6. **Is one-time financial support for securing a flat or a property motivational for you to decide to work in rural areas?** Yes – No
7. **Is one-time financial support for reconstruction or equipment of the surgery motivational for you to decide to work in rural areas?** Yes – No
8. **Would securing a qualified job for your partner in a rural area increase the chance of you working there too?** Yes – No
9. **If finance were the main issue that would influence your decision to work in rural areas, what bonuses would be motivational for you?**
  Bonuses´ structure
  ……………………………………………… Amount …………………………………………
10. **Where do you see obstacles stopping you from working in a rural area?** (Select the five most important factors)
  - Increased amount of work
  - Work interfering with your leisure time (being asked for help outside your office hours)
  - Lack of subsequent secondary care
  - Paperwork
  - Life in the country with its specifics
  - Lesser accessibility of schools for your children
  - Lesser accessibility of jobs for your partner
  - The solitude in your office (feelings of isolation)
  - Others – specify: ………………………….
11. **Which factors do you find motivating to work in a rural area** (Select the five most important factors)?
  - Environmental issues
  - Permanent and continuous contact with people (including times outside the office hours)
  - Long-term nature of care (monitoring individuals or families for up to decades)
  - The doctor’s role in the local community
  - Diversity of work, performing a higher number of tasks, the necessity of being self-reliant
  - Closer relationships
  - Availability of leisure time activities (e.g. gardening, raising animals, gamekeeping, etc.)
  - Availability of leisure time activities for your family (e.g. gardening, raising animals, gamekeeping, etc.)
  - Others – specify: ………………………….
12. **Did you have access to information on rural healthcare during your studies at medical school or during your GP training?** Yes – No
13. **Would you appreciate a short-term training period in a rural practice as part of the GP training programme?** Yes – No
14. **Is there anything you would like to share that could increase your interest in a rural practice?**
